# Genetic Ablation of STE20-Type Kinase MST4 Does Not Alleviate Diet-Induced MASLD Susceptibility in Mice

**DOI:** 10.3390/ijms25042446

**Published:** 2024-02-19

**Authors:** Mara Caputo, Emma Andersson, Ying Xia, Wei Hou, Emmelie Cansby, Max Erikson, Dan Emil Lind, Bengt Hallberg, Manoj Amrutkar, Margit Mahlapuu

**Affiliations:** 1 Department of Chemistry and Molecular Biology, University of Gothenburg and Sahlgrenska University Hospital, 41345 Gothenburg, Sweden; 2 Department of Medical Biochemistry and Cell Biology, Institute of Biomedicine, University of Gothenburg and Sahlgrenska University Hospital, 41345 Gothenburg, Sweden; 3 Department of Pathology, Oslo University Hospital Rikshospitalet, 0372 Oslo, Norway

**Keywords:** MST4, metabolic dysfunction-associated steatotic liver disease, metabolic dysfunction-associated steatohepatitis, liver lipid metabolism, intrahepatocellular lipid droplets

## Abstract

Metabolic dysfunction-associated steatotic liver disease (MASLD) and its advanced subtype, metabolic dysfunction-associated steatohepatitis (MASH), have emerged as the most common chronic liver disease worldwide, yet there is no targeted pharmacotherapy presently available. This study aimed to investigate the possible in vivo function of STE20-type protein kinase MST4, which was earlier implicated in the regulation of hepatocellular lipotoxic milieu in vitro, in the control of the diet-induced impairment of systemic glucose and insulin homeostasis as well as MASLD susceptibility. Whole-body and liver-specific *Mst4* knockout mice were generated by crossbreeding conditional *Mst4*^fl/fl^ mice with mice expressing Cre recombinase under the *Sox2* or *Alb* promoters, respectively. To replicate the environment in high-risk subjects, *Mst4*^–/–^ mice and their wild-type littermates were fed a high-fat or a methionine–choline-deficient (MCD) diet. Different in vivo tests were conducted in obese mice to describe the whole-body metabolism. MASLD progression in the liver and lipotoxic damage to adipose tissue, kidney, and skeletal muscle were analyzed by histological and immunofluorescence analysis, biochemical assays, and protein and gene expression profiling. In parallel, intracellular fat storage and oxidative stress were assessed in primary mouse hepatocytes, where MST4 was silenced by small interfering RNA. We found that global MST4 depletion had no effect on body weight or composition, locomotor activity, whole-body glucose tolerance or insulin sensitivity in obese mice. Furthermore, we observed no alterations in lipotoxic injuries to the liver, adipose, kidney, or skeletal muscle tissue in high-fat diet-fed whole-body *Mst4*^–/–^ vs. wild-type mice. Liver-specific *Mst4*^–/–^ mice and wild-type littermates displayed a similar severity of MASLD when subjected to an MCD diet, as evidenced by equal levels of steatosis, inflammation, hepatic stellate cell activation, fibrosis, oxidative/ER stress, and apoptosis in the liver. In contrast, the in vitro silencing of MST4 effectively protected primary mouse hepatocytes against ectopic lipid accumulation and oxidative cell injury triggered by exposure to fatty acids. In summary, these results suggest that the genetic ablation of MST4 in mice does not mitigate the initiation or progression of MASLD and has no effect on systemic glucose or insulin homeostasis in the context of nutritional stress. The functional compensation for the genetic loss of MST4 by yet undefined mechanisms may contribute to the apparent discrepancy between in vivo and in vitro phenotypic consequences of MST4 silencing.

## 1. Introduction

Metabolic dysfunction-associated steatotic liver disease (MASLD), previously referred to as non-alcoholic fatty liver disease (NAFLD), is defined by fatty infiltration in >5% of hepatocytes after the exclusion of secondary causes (e.g., excessive alcohol consumption, medications, and certain heritable conditions) [1,2,3]. Being closely linked with obesity and type 2 diabetes, MASLD currently affects approximately 30% of the global population, and its prevalence is rapidly rising worldwide [4,5,6,7]. In parallel, metabolic dysfunction-associated steatohepatitis (MASH, formerly known as non-alcoholic steatohepatitis (NASH)), which is the more active subtype of MASLD characterized by hepatic steatosis, inflammation, cell damage in the form of ballooning, and different stages of fibrosis, is emerging as one of the major catalysts for cirrhosis, hepatocellular carcinoma (HCC), and liver-related death [8,9,10,11,12,13]. Mechanistically, elevated lipid deposition within hepatocytes during the early stages of MASLD causes the chronic activation of oxidative and endoplasmic reticulum (ER) stress, which provokes local inflammation, fibrinogenesis, and apoptosis, ultimately driving the disease progression to MASH [8,14,15,16,17]. Thus, deciphering the pathways that control intrahepatocellular lipid storage and metabolic stress is highly relevant to understanding the molecular pathogenesis of the initiation and aggravation of MASLD.

In search for new proteins that govern susceptibility to MASLD and MASH, we recently pinpointed STE20-type kinase MST4 (mammalian sterile 20 like 4; also known as STK26 or MASK) as a novel controller of hepatocellular lipid accumulation and related cell damage under conditions of nutritional stress. We found that *MST4* mRNA abundance in human liver biopsies positively correlates with the severity of MASLD (i.e., hepatic steatosis, lobular inflammation, and ballooning) [18]. Moreover, we observed that MST4 knockdown in cultured human hepatocytes effectively alleviates excessive lipid deposition by eliciting a shift from lipid anabolism (fatty acid uptake and lipogenesis) towards lipid catabolism (mitochondrial β-oxidation and triacylglycerol (TAG) secretion) [18]. Conversely, the overexpression of MST4 aggravates hepatocellular fat storage by reducing β-oxidation and TAG efflux while stimulating lipid synthesis [18]. We also demonstrated that the knockdown or overexpression of MST4 in human hepatocytes decreases or increases oxidative and ER stress, respectively [18]. Interestingly, our studies revealed that the MST4 protein binds to intrahepatocellular lipid droplets [18], which is consistent with a new unified view of these complex organelles as critical regulators of the energy homeostasis of the cell [19,20,21]. Of note, we observed that hepatic *MST4* levels positively correlate with the incidence and severity of MASH-driven HCC and that the depletion of MST4 markedly suppresses the proliferative, migratory, and invasive capacity as well as the epithelial–mesenchymal transition of human HCC cell lines [22]. Recent studies have also implicated MST4 in the progression of several other cancer types [23,24,25,26,27,28,29,30]. In addition, MST4 has been linked to the modulation of neuronal function [31] and immune responses [32,33], as well as the pathology of endothelial malformations [34,35]. 

Based on our earlier mechanistic studies in cultured hepatocytes, which suggest a role for MST4 in the regulation of hepatocellular lipotoxicity in vitro, we here applied the genetic models of whole-body and liver-specific *Mst4* knockout mice to investigate the potential in vivo function of this kinase in the diet-induced impairment of systemic glucose and insulin homeostasis as well as MASLD susceptibility. 

## 2. Results

### 2.1. Global Knockout of MST4 in Obese Mice Has No Effect on Body Weight or Composition, Locomotor Activity, or Glucose or Insulin Homeostasis 

**Figure 1:**
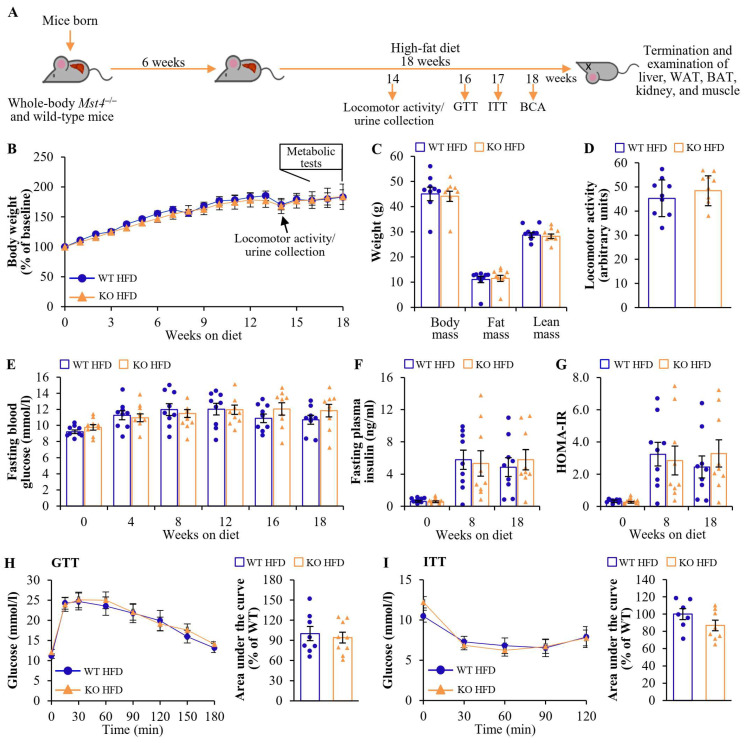
Global knockout of MST4 does not affect body weight or composition, locomotor activity, or glucose or insulin homeostasis in high-fat diet-fed mice. (**A**) Schematic illustration of the experimental design. (**B**) Body weight curves. (**C**) Total, fat, and lean body mass were measured using BCA. (**D**) Locomotor activity was assessed via the open-field test. (**E**,**F**) Fasting circulating levels of glucose (**E**) and insulin (**F**). (**G**) HOMA-IR was calculated using the equation (fasting glucose (mg/dL) × fasting insulin (ng/mL))/405. (**H**,**I**) Intraperitoneal GTT (**H**) and ITT (**I**); the area under the glucose curve in both tests is shown. Data are the mean ± SEM from 7 to 9 mice per group. HFD, high-fat diet; KO, knockout; WT, wild-type.

To investigate the potential functional role of MST4 on the progression of diet-induced obesity, we challenged male mice with the whole-body depletion of MST4 and their wild-type littermates with a high-fat diet (45 kcal% fat) for 18 weeks, which mimics the environment in high-risk individuals (Figure 1A). *Mst4* knockout mice were born at the expected Mendelian ratio and showed no abnormalities during gross inspection. No alterations in body weight were found between the two groups during the period of the high-fat diet challenge, and the total, fat, and lean body mass analyzed using body composition analysis (BCA) were also comparable in *Mst4*^–/–^ mice and wild-type controls (Figure 1B,C). Liver weight, as well as the weights of adipose tissue depots estimated in absolute values, and when related to total body weight, were similar in *Mst4*^–/–^ vs. wild-type mice (Supplementary Table S2). Open-field tests did not reveal any change in the locomotor activity between the two groups (Figure 1D). 

We detected no alterations in fasting circulating glucose or insulin or the homeostasis model assessment score of insulin resistance (HOMA-IR), measured at several time points during the period of high-fat diet consumption when comparing *Mst4*^–/–^ mice and wild-type controls (Figure 1E–G). The genetic ablation of MST4 had no effect on systemic glucose tolerance or insulin sensitivity, as evidenced by similar outcomes of intraperitoneal glucose tolerance test (GTT) and insulin tolerance test (ITT) performed following 16 and 17 weeks of the high-fat diet challenge, respectively (Figure 1H,I). Notably, we found no difference in the peak insulin concentration during the GTT between the two groups.

### 2.2. Whole-Body Depletion of MST4 Does Not Affect High-Fat Diet-Induced Steatotoxicity in the Liver

**Figure 2:**
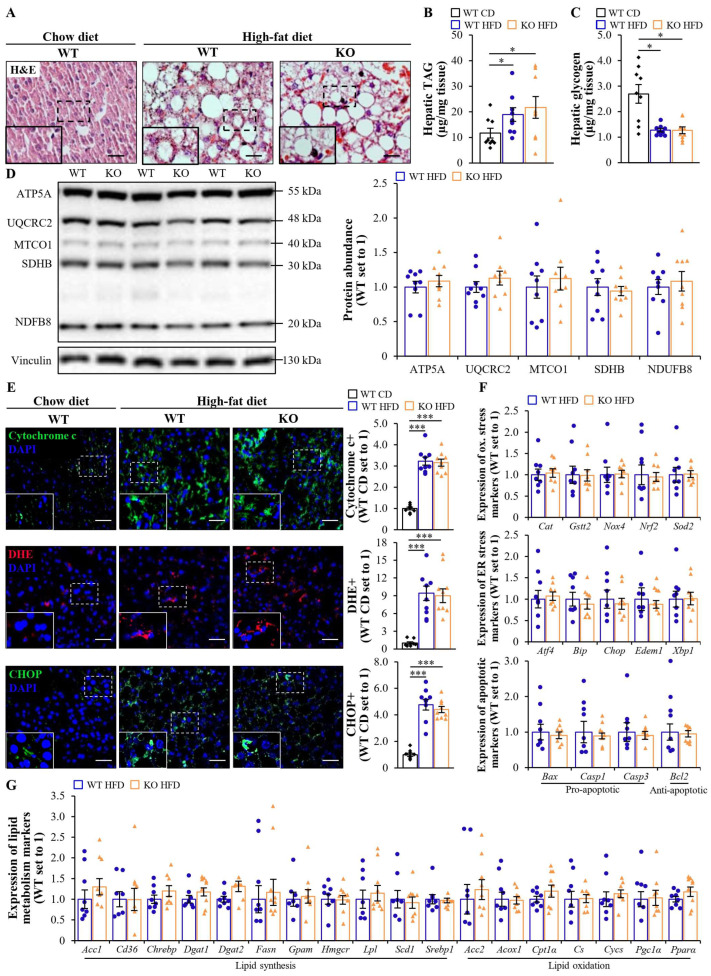
Whole-body depletion of MST4 does not protect mice against high-fat diet-induced steatotoxicity in the liver. (**A**) Representative liver sections were stained with H&E. The scale bars represent 25 µm. (**B**,**C**) The quantification of hepatic TAG (**B**) and glycogen (**C**) content. (**D**) Liver lysates were analyzed via Western blot using an anti-total OXPHOS antibody cocktail. Protein levels were measured using densitometry; representative Western blots are shown with vinculin used as a loading control. (**E**) Representative liver sections were stained with DHE (red) or processed for immunofluorescence with anti-cytochrome c or anti-CHOP (green) antibodies; nuclei were stained with DAPI (blue). The scale bars represent 25 µm. Quantification of the staining. (**F**,**G**) The relative mRNA expression of selected genes controlling oxidative/ER stress and apoptosis (**F**), as well as lipid metabolism (**G**), was assessed via qRT-PCR in the liver. Data are the mean ± SEM from 8 to 9 mice per group. CD, chow diet; HFD, high-fat diet; KO, knockout; ox., oxidative; WT, wild-type. * *p* < 0.05, *** *p* < 0.001.

To assess the possible contribution of MST4 to hepatic steatotoxicity in the context of obesity, we examined ectopic lipid accumulation, mitochondrial activity, and oxidative/ER stress in the livers of high-fat diet-fed mice with the whole-body deletion of MST4 vs. their wild-type littermates. The morphological examination of hematoxylin and eosin (H&E)-stained liver sections did not demonstrate any alterations in micro- or macrovesicular steatosis in *Mst4*^–/–^ mice compared with wild-type controls (Figure 2A). Moreover, no difference was detected in the hepatic TAG or glycogen content between the two groups (Figure 2B,C). MST4 deficiency had no impact on the liver mitochondrial function as evidenced by comparable protein levels of essential components in the oxidative phosphorylation (OXPHOS) pathway (key enzymes comprising the electron transport chain and ATP synthase in mitochondria) and a similar abundance of cytochrome c (an electron-carrying mitochondrial protein) in both genotypes (Figure 2D,E). The liver sections from *Mst4*^–/–^ and wild-type mice also displayed equal levels of oxidative stress quantified by dihydroethidium (DHE) staining for superoxide radicals (O_2_^•−^) and ER stress monitored by immunostaining for the KDEL (a signal motif for ER retrieval) and C/EBP-homologous protein (CHOP; a marker for ER stress-mediated cell death) (Figure 2E; Supplementary Figure S2). Consistently, the hepatic mRNA expression of oxidative/ER stress and apoptotic markers, as well as key regulators of lipid metabolism, was equivalent between the genotypes (Figure 2F,G). 

Of note, the whole-body deletion of MST4 had no effect on the hepatic phosphorylation of acetyl-CoA carboxylase (ACC; a critical mediator of both lipid synthesis and oxidation) or Jun N-terminal kinase (JNK; a key controller of mitochondrial function), or the conversion of LC3-I to LC3-II (a parameter indicating autophagic flux) (Supplementary Figure S3). 

### 2.3. Global Deficiency of MST4 Has No Impact on Adipose Tissue Function or Renal or Skeletal Muscle Lipotoxicity in High-Fat Diet-Fed Mice

**Figure 3:**
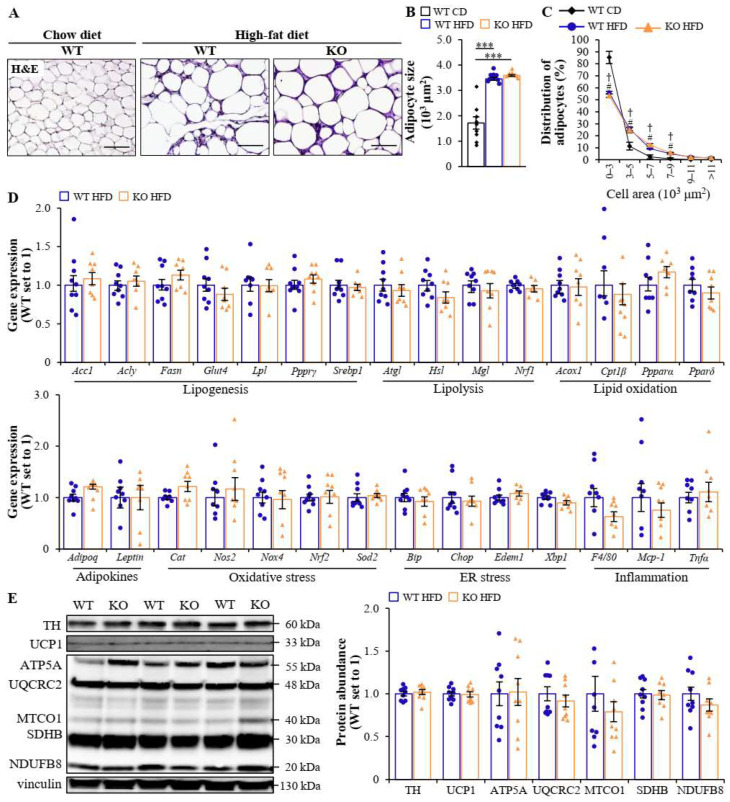
Global deficiency of MST4 has no effect on adipose tissue function in obese mice. (**A**) Representative eWAT sections were stained with H&E. The scale bars represent 100 µm. (**B**,**C**) The average adipocyte size (**B**) and adipocyte size distribution with values representing the relative proportion of adipocytes in the given diameter class (**C**) in the eWAT. (**D**) Relative mRNA expression of adipokines and selected genes controlling lipid metabolism, oxidative/ER stress, and inflammation was assessed via qRT-PCR in the sWAT. (**E**) BAT lysates were analyzed via Western blot using antibodies specific for TH or UCP1, or applying the anti-total OXPHOS antibody cocktail. Protein levels were measured using densitometry; representative Western blots are shown with vinculin used as a loading control. Data are the mean ± SEM from 7 to 9 mice per group. CD, chow diet; HFD, high-fat diet; KO, knockout; WT, wild-type. *** *p* < 0.001 for mice fed a high-fat vs. chow diet; ^†^ *p* < 0.001 for wild-type mice fed a high-fat vs. chow diet; and ^#^ *p* < 0.001 for *Mst4*^–/–^ mice fed a high-fat vs. chow diet.

In line with the equal fat mass in both genotypes (Figure 1C), we observed a similar adipocyte size in the epididymal white adipose tissue (eWAT) from obese *Mst4* knockout and wild-type mice (Figure 3A–C). Consistently, the depletion of MST4 had no impact on the transcript levels of adipokines or genes mediating lipid metabolism, oxidative/ER stress, or inflammation in the subcutaneous white adipose tissue (sWAT) (Figure 3D). Furthermore, the protein levels of tyrosine hydroxylase (TH; a sympathetic neuron indicator), uncoupling protein 1 (UCP1; a mitochondrial protein uncoupling cellular respiration to dissipate energy in the form of heat), and key enzymes in the OXPHOS pathway were comparable in the brown adipose tissue (BAT) from *Mst4^–/–^* mice and wild-type controls (Figure 3E).

**Figure 4:**
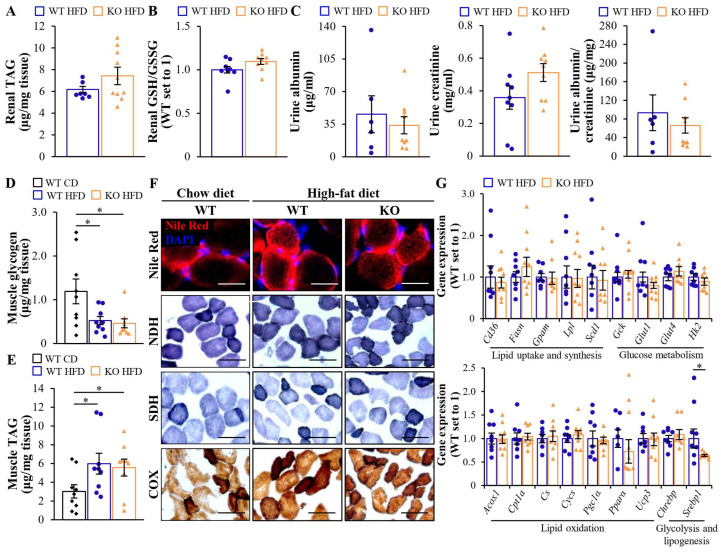
The whole-body depletion of MST4 has no effect on renal or skeletal muscle lipotoxicity in obese mice. (**A**,**B**) Assessment of TAG content (**A**) and the ratio of GSH/GSSH (**B**) in kidney lysates. (**C**) Measurement of urinary albumin, creatinine, and albumin-to-creatinine ratio. (**D**,**E**) Quantification of glycogen (**D**) and TAG (**E**) in gastrocnemius skeletal muscle lysates. (**F**) Representative gastrocnemius skeletal muscle sections were stained with Nile Red (red), nuclei were stained with DAPI (blue), or processed in enzymatic activity assays for NDH, SDH, or COX. The scale bars represent 25 µm. (**G**) The relative mRNA expression of selected genes controlling lipid and glucose metabolism was assessed by qRT-PCR in gastrocnemius skeletal muscle. Data are the mean ± SEM from 6 to 9 mice per group. CD, chow diet; KO, knockout; WT, wild-type. * *p* < 0.05.

To investigate diet-induced lipotoxicity in the kidney, we analyzed ectopic fat storage and antioxidant capacity in the renal lysates from high-fat diet-fed *Mst4* knockout mice vs. wild-type littermates. We found an equal amount of TAG and a comparable ratio of reduced-to-oxidized glutathione (GSH/GSSG) between the two groups (Figure 4A,B). Furthermore, albuminuria assessed as the urinary albumin-to-creatinine ratio was similar in both genotypes (Figure 4C). 

We observed equal glycogen levels in the gastrocnemius skeletal muscle from obese *Mst4^–/–^* and wild-type mice (Figure 4D). Intramyocellular fat storage, assessed by measuring the TAG content in the muscle lysates and staining the muscle sections with the lipophilic dye Nile Red, was also equivalent when comparing the two groups (Figure 4E,F). The genetic ablation of MST4 did not affect the activity of NADH dehydrogenase (NDH), succinate dehydrogenase (SDH), or cytochrome c oxidase (COX) in the skeletal muscle, which represent the complexes in the mitochondrial electron transport chain (Figure 4F). Moreover, the mRNA abundance of proteins regulating lipid and glucose metabolism was similar in the skeletal muscle collected from the two groups (Figure 4G). 

### 2.4. Liver-Specific Deletion of MST4 Does Not Protect Mice against MCD Diet-Induced MASH

**Figure 5:**
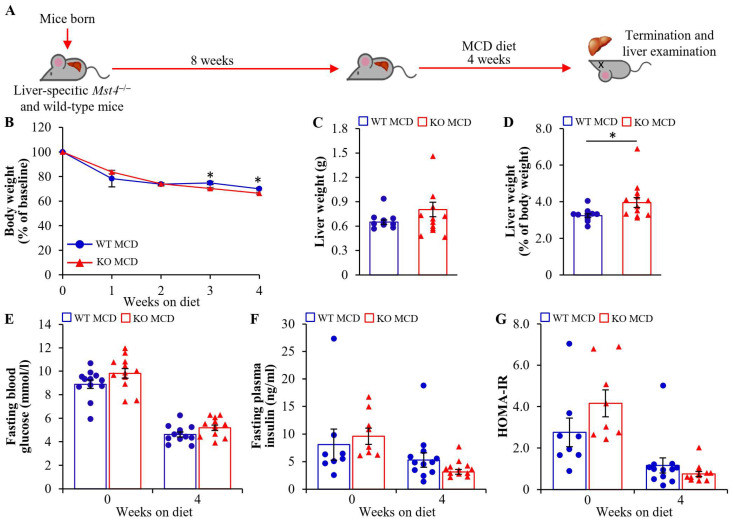
Liver-specific ablation of MST4 has no impact on systemic glucose or insulin homeostasis in MCD diet-fed mice. (**A**) Schematic illustration of the experimental design. (**B**–**D**) Body weight curves (**B**), liver weight estimated in absolute values (**C**), and when related to total body weight (**D**). (**E**,**F**) Fasting circulating levels of glucose (**E**) and insulin (**F**). (**G**) HOMA-IR was calculated using the equation (fasting glucose (mg/dL) × fasting insulin (ng/mL))/405. Data are the mean ± SEM from 8 to 12 mice per group. KO, knockout; WT, wild-type. * *p* < 0.05.

To examine the potential function of MST4 in the progression of MASLD to MASH, male mice with the liver-specific depletion of MST4 and their wild-type littermates were exposed to a high-carbohydrate diet lacking methionine and choline (MCD) for 4 weeks, which mimics the main pathophysiological features of human MASH, including liver steatosis, inflammation, fibrosis, and hepatocellular injury (Figure 5A). As expected, a marked down-regulation in body weight by the MCD diet challenge was found in both groups, with a minor difference between the genotypes (about 5% lower in *Mst4* knockout mice; Figure 5B). Liver weight estimated in absolute values was comparable between the two groups, whereas the liver weight related to total body weight was slightly increased in *Mst4*^–/–^ vs. wild-type mice (Figure 5C,D). The levels of blood glucose, plasma insulin, and HOMA-IR decreased in both groups in response to MCD diet feeding but remained similar when comparing the two genotypes (Figure 5E–G). 

**Figure 6:**
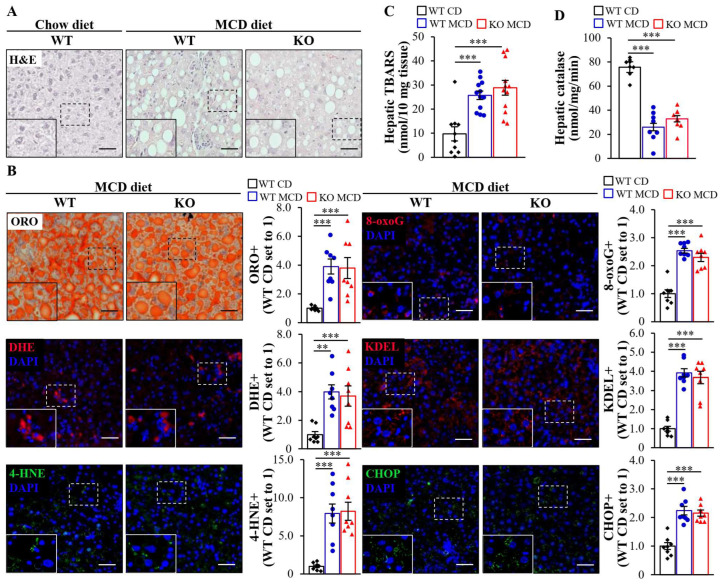
Liver-specific inhibition of MST4 does not protect mice against MCD diet-induced hepatic steatosis or oxidative/ER stress. (**A**) Representative liver sections were stained with H&E. The scale bars represent 25 µm. (**B**) Representative liver sections were stained with Oil Red O or DHE (red) or processed for immunofluorescence with anti-4-HNE (green), anti-8-oxoG (red), anti-KDEL (red), or anti-CHOP (green) antibodies; nuclei were stained with DAPI (blue). The scale bars represent 25 µm. Quantification of the staining. (**C**,**D**) Measurement of TBARS (**C**) and catalase activity (**D**) in the liver lysates. Data are the mean ± SEM from 7 to 9 mice per group. Data of Oil Red O-stained area in chow diet-fed mice were extracted from the previous study [36]. CD, chow diet; KO, knockout; ORO, Oil Red O; WT, wild-type. ** *p* < 0.01, *** *p* < 0.001.

**Figure 7:**
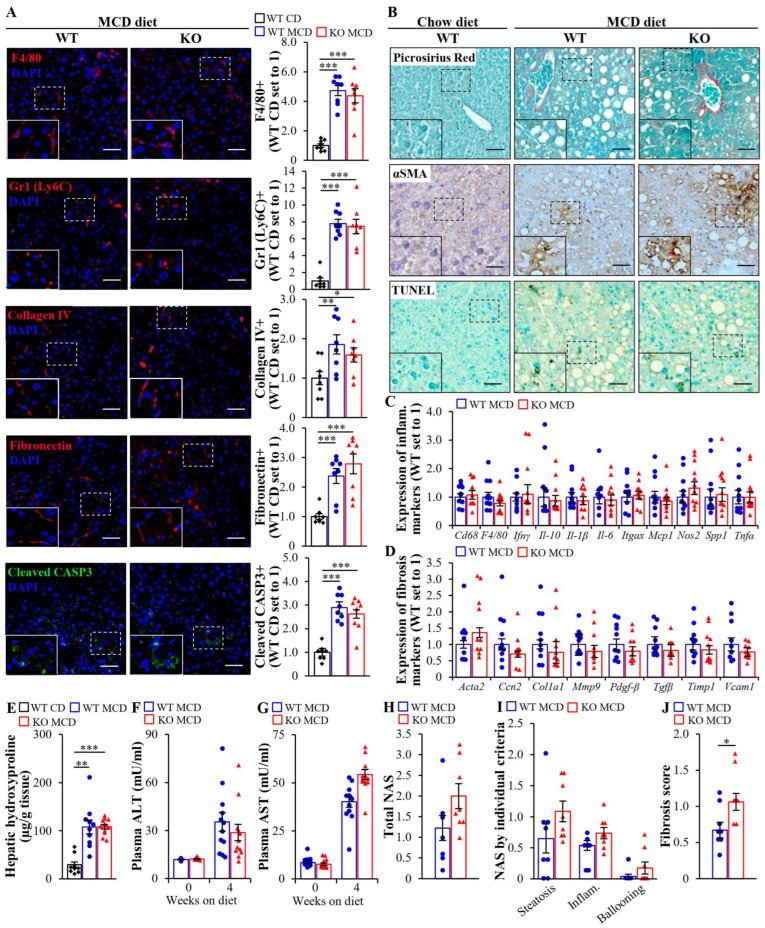
Liver-specific *Mst4*^–/–^ mice are not protected against MCD diet-induced hepatic inflammation or fibrosis. (**A**) Representative liver sections were processed for immunofluorescence with anti-F4/80, anti-Gr1 (Ly6C), anti-collagen IV, anti-fibronectin (red), or anti-cleaved CASP3 (green) antibodies; nuclei stained with DAPI (blue). The scale bars represent 25 µm. Quantification of the staining. (**B**) Representative liver sections were stained with Picrosirius Red or TUNEL, or processed for immunohistochemistry with anti-αSMA antibodies; they were counterstained with Fast Green (Picrosirius Red), methyl green (TUNEL), or hematoxylin (αSMA). The scale bars represent 25 µm. (**C**,**D**) Relative mRNA expression of selected genes controlling inflammation (**C**) and fibrosis (**D**) was assessed via qRT-PCR in the liver. (**E**) Quantification of hepatic hydroxyproline. (**F**,**G**) Measurement of plasma ALT (**F**) and AST (**G**) activity. (**H**,**I**) Assessment of total NAS (**H**) and individual histological features of NAS (steatosis 0–3, inflammation 0–3, hepatocellular ballooning 0–2) (**I**) in H&E-stained liver sections. (**J**) The fibrosis score was assessed based on the Kleiner/Brunt criteria adapted to rodents (0, no fibrosis; 1, focal pericellular fibrosis in zone 3; 2, perivenular and pericellular fibrosis confined to zones 2 and 3; 3, bridging fibrosis; and 4, cirrhosis) in the liver sections stained with Picrosirius Red and counterstained with Fast Green. Data are the mean ± SEM from 8 to 12 mice per group. CD, chow diet; inflam., inflammation; KO, knockout; WT, wild-type. * *p* < 0.05, ** *p* < 0.01, *** *p* < 0.001.

Next, we assessed the severity of hepatic steatosis, inflammation, and fibrosis in MCD diet-challenged *Mst4*^–/–^ and wild-type mice. The staining of liver sections with H&E or lipophilic dye Oil Red O showed a similar amount of micro- and macrovascular steatosis in *Mst4*^–/–^ mice and wild-type littermates (Figure 6A,B). Furthermore, the depletion of MST4 had no impact on hepatic oxidative stress quantified by DHE staining for superoxide radicals and the immunofluorescence analysis of 4-hydroxynonenal (4-HNE) and 8-oxoguanine (8-oxoG), which detect lipid peroxidation products and oxidative DNA damage, respectively (Figure 6B). Consistently, the concentration of thiobarbituric acid-relative substance (TBARS) (a classic indicator of lipid peroxidation) and the enzymatic activity of catalase (a marker of antioxidant defense) were not altered in the liver lysates from *Mst4*^–/–^ vs. wild-type mice (Figure 6C,D). The levels of hepatic ER stress examined by immunostaining for KDEL and CHOP were also equivalent between the genotypes (Figure 6B). MST4 abrogation did not affect the hepatic abundance of Kupffer cells or monocyte-derived macrophages, as evidenced by comparable numbers of F4/80- and Gr1 (Ly6C)-positive cells in the livers from *Mst4*^–/–^ and wild-type mice. In addition, no change in hepatic fibrosis was observed in *Mst4* knockout mice as demonstrated by similar labeling for liver collagen IV, fibronectin, Picrosirius Red (stains both collagen type I and type III), and αSMA (a marker for activated hepatic stellate cells, which secrete extracellular matrix proteins) in the two groups (Figure 7A,B). In line with immunostainings, we found no alternations in the expression of mRNA indicators of inflammation or fibrosis in the livers from *Mst4*^–/–^ vs. wild-type mice (Figure 7C,D). Consistently, the depletion of MST4 had no effect on the hepatic hydroxyproline content (an indicator of liver collagen deposition) (Figure 7E) or hepatocellular apoptosis (assessed via terminal deoxynucleotidyl transferase-mediated deoxyuridine triphosphate nick-end labeling (TUNEL) and immunostaining for cleaved caspase 3 (CASP3)) (Figure 7A,B).

The measurement of alanine aminotransferase (ALT) and aspartate aminotransferase (AST) activity (the most commonly used plasma biomarkers of MASH) did not indicate any change in MCD diet-fed *Mst4*^–/–^ vs. wild-type mice (Figure 7F,G). The MASH severity evaluated by total NAFLD activity score (NAS), as well as its individual components, i.e., histological liver steatosis, lobular inflammation, and hepatocellular ballooning scores, was also similar between the genotypes (Figure 7H,I). Notably, an increase in the histological fibrosis score was detected in the livers of *Mst4*^–/–^ mice compared to wild-type controls (Figure 7J). However, since hepatic fibrosis markers were not elevated in *Mst4* knockout mice, this observation should be interpreted as an incidental finding with no clear explanation.

While we found no difference in the severity of MASH in MCD diet-fed *Mst4*^–/–^ mice vs. wild-type littermates, we observed that both genotypes challenged by an MCD diet developed marked liver steatosis, inflammation, cell damage, and fibrosis compared with age-matched wild-type mice fed a control (chow) diet, which serve as a reference group in most assays (Figure 6 and Figure 7; Supplementary Figure S4).

### 2.5. Possible Mechanism behind the Absence of Hepatic Phenotype in Mst4 Knockout Mice

**Figure 8:**
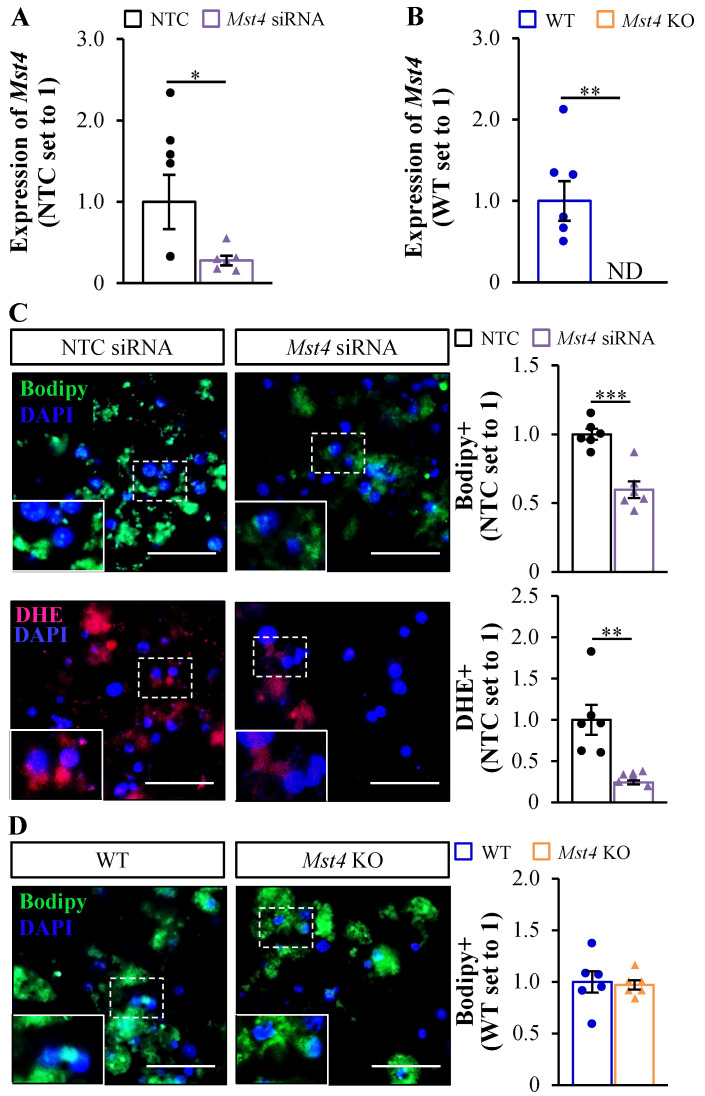
The transfection of primary mouse hepatocytes with *Mst4* siRNA suppresses lipotoxicity when compared with cells transfected with NTC siRNA, which is not replicated in hepatocytes derived from *Mst4*^–/–^ vs. wild-type mice. (**A**,**C**) Primary hepatocytes were isolated from wild-type mice, transfected with mouse *Mst4* siRNA or NTC siRNA, and cultured with oleate supplementation. (**A**) The relative mRNA expression of *Mst4* was assessed via qRT-PCR. (**C**) Representative images of hepatocytes stained with Bodipy 493/503 (green) or DHE (red); nuclei were stained with DAPI (blue). The scale bars represent 25 µm. Quantification of the staining. (**B**,**D**) Primary hepatocytes were isolated from whole-body *Mst4*^–/–^ and wild-type mice and cultured with oleate supplementation. (**B**) The relative mRNA expression of *Mst4* was assessed via qRT-PCR. In (**B**), statistical significance between the groups was evaluated using the nonparametric Kruskal–Wallis test, followed by Dunn’s multiple comparison test. (**D**) Representative images of hepatocytes stained with Bodipy 493/503 (green); nuclei were stained with DAPI (blue). The scale bars represent 25 µm. Quantification of the staining. Data are the mean ± SEM from 5 to 6 wells per group. KO, knockout; ND, not detected; WT, wild-type. * *p* < 0.05, ** *p* < 0.01, *** *p* < 0.001.

In this study, we detected no change in diet-induced liver steatosis or related lipotoxic damage in *Mst4*^–/–^ vs. wild-type mice, which conflicts with our previous reports describing the critical function of MST4 in provoking ectopic fat deposition and oxidative/ER stress in cultured human hepatocytes [18]. To examine whether this disparity could potentially arise from species-specific differences in the function of the human vs. mouse *MST4* gene, we investigated the effect of MST4 knockdown in vitro in primary hepatocytes isolated from wild-type mice. We observed that similarly to our results obtained in human MST4-deficient hepatocytes [18], the mouse hepatocytes transfected with *Mst4* small interfering (si)RNA displayed a significantly suppressed lipid content and oxidative stress as evidenced by lower staining for Bodipy 493/503 and DHE, respectively, compared with cells transfected with non-targeting control (NTC) siRNA (Figure 8A,C). Notably, consistent with our findings in the liver sections and lysates from *Mst4* knockout mice, we detected no decrease in lipid deposition in primary hepatocytes derived from *Mst4*^–/–^ vs. wild-type mice (Figure 8B,D). Together, these observations indicate that the discrepancy in the phenotypic consequences of MST4 inactivation in the mouse liver vs. human hepatocytes is not caused by species-specific divergence but rather reflects the difference between in vivo vs. in vitro experimental design.

## 3. Discussion

In the current study, we examined the in vivo function of the STE20-type protein kinase MST4 in systemic metabolic impairment and MASLD progression based on the phenotypic characterization of global and liver-specific *Mst4* knockout mice challenged with a high-fat or an MCD diet, respectively. We found that high-fat diet-fed whole-body *Mst4*^–/–^ mice displayed equal levels of lipotoxic damage to the liver as well as extrahepatic tissues when compared with wild-type littermates, which was accompanied by a similar degree of systemic glucose intolerance and insulin resistance in both genotypes. Our results also revealed that the liver-specific deletion of MST4 had no effect on the development of MCD diet-induced MASH in mice.

Our earlier studies demonstrated that hepatic *MST4* expression positively correlates with the severity of human MASLD and that the in vitro silencing of MST4 in human hepatocytes markedly suppresses lipotoxicity triggered by exposure to fatty acids [18,22]. In light of this evidence, it was unexpected that we did not detect any in vivo phenotypic effect of the genetic loss of MST4 on diet-induced liver steatotoxicity, metabolic dysfunction, or MASLD susceptibility in mice. The discrepancy between observations in *Mst4*^–/–^ mice and our previous investigations in human liver biopsies and MST4-deficient human hepatocytes does not seem to be caused by species-specific differences in gene function, as we found lower fat storage and reduced oxidative stress even in cultured mouse hepatocytes, where MST4 was knocked down using siRNA. Consistently, MST4 is highly conserved in mice and humans, with an amino-acid sequence identity of 98%. 

Alternatively, the lack of hepatic alterations in *Mst4*^–/–^ mice could potentially be attributed to the ability of related STE20-type kinases to functionally compensate for the genetic loss of MST4. To this end, different STE20 family proteins have recently been implicated in exacerbating the risk of MASLD in the context of obesity. Similarly to MST4 [18], STK25 and MST3 (comprising the GCK-III subfamily of STE20 kinases together with MST4), MAP4K4 (belonging to the GCK-IV subfamily), as well as TAOK1 and TAOK3 (GCK-VIII subfamily members) have been identified as important drivers of hepatocellular lipotoxicity based on expression profiling in human liver biopsies, which demonstrates that their mRNA abundance is positively correlated with the key histological features of MASLD, and via in vitro investigations in cultured hepatocytes, which reveal that the knockdown of their respective genes alleviates fat accumulation by causing a shift from lipid anabolism towards catabolism [18,36,37,38,39,40,41]. We and other research groups also found that the inactivation of STK25 or MST3 in mice, either by genetic knockout or treatment with antisense oligonucleotides, improves whole-body insulin sensitivity and effectively ameliorates the full spectrum of diet-induced MASLD encompassing suppressed hepatic steatosis, inflammation, and fibrosis [36,42,43,44]. Notably, all the STE20 kinases listed above, except MAP4K4, are components of the hepatocellular lipid droplet proteome, thus co-localizing with MST4 [36,37,39]. On the basis of this evidence, we speculate that the related STE20-type kinases, which share the subcellular localization pattern and carry out overlapping functions with MST4 in hepatocytes, have the potential to functionally compensate for the lack of MST4 in knockout mice, where this gene has been depleted throughout development, but not in MST4-deficient hepatocytes cultured in vitro, where the gene function may be inhibited by siRNA before any compensatory mechanisms can be triggered. We did not, however, detect any up-regulation of the mRNA expression or protein abundance of STK25, MST3, MAP4K4, TAOK1, or TAOK3 when comparing the livers from *Mst4*^–/–^ mice and wild-type controls (Supplementary Figure S5). Importantly, the cellular mode-of-action of MST4 and related STE20-type kinases remains largely elusive, and the further characterization of their upstream activators, interaction partners, and downstream substrates is critical to understanding the shared vs. unique functions as well as synergies and redundancies in this complex family of key metabolic regulators. 

In summary, our findings reveal that the genetic abrogation of MST4 in mice fails to improve whole-body glucose tolerance or insulin sensitivity or mitigate the development of MASLD under conditions of nutritional stress. This contrasts with the protective response against lipotoxic damage described by the silencing of MST4 in human and mouse hepatocytes in vitro and may be attributable to the functional compensation for the genetic loss of MST4 by yet undefined mechanisms.

## 4. Materials and Methods

### 4.1. Animal Experiments

Whole-body and liver-specific *Mst4* knockout mice were generated by crossbreeding conditional *Mst4*^fl/fl^ mice (MGI ID 1917665; Institute of Molecular Genetics of the Czech Academy of Sciences, Prague, Czech Republic) with mice expressing Cre recombinase under *Sox2* (stock no. 008454; Jackson Laboratory, Bar Harbor, ME, USA) or *Alb* (stock no. 003574; Jackson Laboratory) promoters, respectively (all on the C57BL/6 J background; Supplementary Figure S1A). The depletion of exons 5 to 8 and flanking introns was verified via the sequencing of DNA (Eurofins Genomics, Ebersberg, Germany) as well as a PCR and quantitative real-time PCR (qRT-PCR) in the liver tissue from the offspring (Supplementary Figure S1B–F). Male *Mst4*^–/–^ mice and their wild-type littermates (Cre-negative mice carrying either the *Mst4* wild-type or floxed allele) were weaned at 3 weeks of age and housed 2–5 per cage in a temperature-controlled (21 °C) facility with ad libitum access to chow diet and water on a 12 h light/dark cycle. From the age of 6 or 8 weeks, the mice were challenged with a pelleted high-fat diet (45 kcal% fat; D12451; Research Diets, New Brunswick, NJ, USA) for 18 weeks or a MCD diet (A02082002B; Research Diets) for 4 weeks, respectively. The body weights were recorded, blood was collected for the determination of glucose and insulin at several time points, and different in vivo tests were conducted, as described below. At the age of 24 (high-fat diet) or 12 weeks (MCD diet), mice were killed by cervical dislocation under isoflurane (Apoteket AB, Stockholm, Sweden) anesthesia after 4 h of food withdrawal. Blood was obtained via cardiac puncture and liver, eWAT, sWAT, BAT, kidney, and gastrocnemius skeletal muscle were harvested for a histological and immunofluorescence microscopy assessment and/or flash frozen in liquid nitrogen and stored at −80 °C for the examination of the protein and gene expression and biochemical analysis, as described below.

The mice utilized in this study received humane care in accordance with the National Institutes of Health (NIH; Bethesda, MD, USA) recommendations outlined in the Guide for the Care and Use of Laboratory Animals. All in vivo experiments were carried out in compliance with the guidelines approved by the local Ethics Committee for Animal Studies at the Administrative Court of Appeals in Gothenburg, Sweden (approval number 5.8.18-17285/2018).

### 4.2. Isolation of Primary Mouse Hepatocytes, Cell Culture, and Transient Transfections

Primary hepatocytes were isolated from the C57BL/6J strain of mice applying a collagenase perfusion method and maintained in Williams E medium (Invitrogen, Carlsbad, CA, USA) supplemented with 0.28 mol/L of sodium ascorbate (Sigma-Aldrich, St. Louis, MO, USA), 0.1 mmol/L of sodium selenite (Sigma-Aldrich), 100 mg/mL of penicillin and 100 U/mL of streptomycin (Gibco, Paisley, UK), 3 g/L of glucose (Sigma-Aldrich), and 26 U/L of human recombinant insulin (Actrapid Penfill; Novo Nordisk, Bagsværd, Denmark). Primary mouse hepatocytes were transfected with mouse *Mst4* siRNA (M-051436-00-0010; Dharmacon, Lafayette, CO, USA) or NTC siRNA (4390844; Invitrogen) using Lipofectamine RNAiMax (Thermo Fisher Scientific, Waltham, MA, USA). Transfected cells were incubated with 50 μmol/L of oleic acid (Sigma-Aldrich) for 48 h prior to harvest.

### 4.3. In Vivo Tests

BCA of total, fat, and lean body mass was performed by time-domain nuclear magnetic resonance (TD-NMR) with the Minispec LF110 Analyzer (Bruker Corporation, Rheinstetten, Germany). Activity was assessed by the open-field test: mice were introduced in the center of a square arena (25 × 25 × 25 cm) to allow free exploration, and locomotor activity was recorded for 15 min during the dark phase of the day over 3 consecutive days and analyzed using the EthoVision XT software (8.5v; Noldus, Wageningen, The Netherlands). Urine was collected over 24 h by using custom-made Perspex restraint cages. GTT or ITT was carried out after 4 h of the morning fast by in the traperitoneal administration of glucose (1 g/kg; Sigma-Aldrich) or human recombinant insulin (1.5 U/kg; Actrapid Penfill; Novo Nordisk), respectively. In vivo tests were conducted only in mice fed a high-fat diet. 

### 4.4. Immunofluorescence and Immunohistochemical Staining

Liver and eWAT tissues were fixed in 4% (*v*/*v*) phosphate-buffered formaldehyde (Histolab Products, Gothenburg, Sweden) and embedded in paraffin, followed by sectioning. Liver sections were stained with H&E (Histolab Products) for morphological examination or with DHE (Life Technologies, Grand Island, NY, USA) to assess superoxide radical formation. Liver sections were also processed for immunofluorescence or immunohistochemistry by incubating the samples with primary antibodies prior to their incubation with fluorescent dye-conjugated or biotinylated secondary antibodies (see Supplementary Table S1 for antibody details). Hepatic apoptotic cells were identified using the TUNEL Assay Kit (Abcam, Cambridge, UK). NAS and fibrosis score were analyzed in liver sections stained with H&E or Picrosirius Red (Histolab Products) and counterstained with Fast Green (Sigma-Aldrich), respectively, according to the Kleiner/Brunt criteria adapted for rodents [45,46,47]. Adipocyte size distribution in eWAT was determined in H&E-stained tissue by a semi-automated analysis using ImageJ software (1.47v; NIH), as previously described [48]. Liver and gastrocnemius skeletal muscle tissues were also embedded in an optimal cutting temperature (OCT) mounting medium (Histolab Products), frozen in liquid nitrogen, and cryo-sectioned. Liver cryosections were stained with Oil Red O (Sigma-Aldrich) to assess the lipid content. Gastrocnemius skeletal muscle cryosections were stained with Nile Red (Sigma-Aldrich) for the determination of lipids or subjected to enzymatic activity assessments, as previously described [49]. Primary mouse hepatocytes were stained with Bodipy 493/503 (Invitrogen) or DHE (Life Technologies) to measure intracellular lipid accumulation or superoxide radical formation, respectively. ImageJ software (1.47v; NIH) was used to quantify the total labeled area in 6 to 10 randomly selected microscope fields (×20) per mouse (distributed over three non-sequential serial sections) or per well of the cell culture chamber.

### 4.5. Biochemical Assays

The blood glucose concentration was assessed using an Accu-Chek glucometer (Roche Diagnostics, Basel, Switzerland). The plasma insulin levels were determined with the Ultra-Sensitive Mouse Insulin ELISA Kit (Crystal Chem, Downers Grove, IL, USA). The TAG content was analyzed in the liver, kidney, and gastrocnemius skeletal muscle lysates using the Triglyceride Colorimetric Assay Kit (Cayman Chemical, Ann Arbor, MI, USA). Glycogen concentration was measured in the liver and gastrocnemius skeletal muscle using the Glycogen Assay Kit (Sigma-Aldrich). The reduced and oxidized glutathione was quantified in kidney lysates using the GSH-Glo Glutathione Assay Kit (Promega, Madison, WI, USA). Urine albumin and creatinine levels were analyzed with the Mouse Albumin ELISA Kit and the Creatinine Assay Kit (both from Abcam), respectively. TBARS concentration and catalase activity were estimated in liver lysates using the Lipid Peroxidation (MDA) Assay Kit (Sigma-Aldrich) and the Catalase Assay Kit (Cayman Chemical), respectively. To investigate collagen content, liver samples were homogenized in dH_2_O and hydrolyzed in 12 N HCl for 3 h at 120 °C, followed by an assessment with the Hydroxyproline Colorimetric Assay Kit (Biovision, Mountain View, CA, USA). ALT and AST levels in plasma were measured using the ALT Activity Assay Kit and the AST Activity Assay Kit (both from Sigma-Aldrich), respectively. All biochemical assays were performed in duplicate.

### 4.6. Western Blot and qRT-PCR

Western blot was conducted as previously described (see Supplementary Table S1 for antibody details) [18]. RNA was extracted from tissue samples and primary mouse hepatocytes using the EZNA Total RNA Kit (Omega Bio-Tek, Norcross, GA, USA) or the RNeasy Lipid Tissue Mini Kit (used for sWAT; Qiagen, Hilden, Germany), followed by reverse transcription with the High-Capacity cDNA Reverse Transcription Kit (Thermo Fisher Scientific). Relative quantification was carried out with the CFX Connect Real-Time System (Bio-Rad, Hercules, CA, USA). The relative abundance of the target transcripts was normalized to the endogenous control, 18S rRNA (Thermo Fisher Scientific).

### 4.7. Statistical Analysis

Statistical comparisons were performed either with unpaired 2-tailed Student’s *t*-test (between two groups) or with 1-way ANOVA followed by a 2-tailed Student’s *t*-test for post hoc analysis (for more than two groups). All statistical analyses were conducted using SPSS statistics (v27; IBM Corporation, Armonk, NY, USA) with *p* < 0.05 considered to be statistically significant.

## Data Availability

The raw data supporting the conclusions of this article will be made available by the authors on request.

## Supplementary Materials

The following supporting information can be downloaded at: https://www.mdpi.com/article/10.3390/ijms25042446/s1.

## Abbreviations

4-HNE: 4-hydroxynonenal; 8-oxoG, 8-oxoguanine; ACC, acetyl-CoA carboxylase; ALT, alanine aminotransferase; AST, aspartate aminotransferase; BAT, brown adipose tissue; BCA, body composition analysis; CHOP, C/EBP-homologous protein; COX, cytochrome c oxidase; DHE, dihydroethidium; ER, endoplasmic reticulum; eWAT, epididymal white adipose tissue; GSH, reduced glutathione; GSSG, oxidized glutathione; GTT, glucose tolerance test; HCC, hepatocellular carcinoma; H&E, hematoxylin and eosin; HOMA-IR, homeostasis model assessment score of insulin resistance; ITT, insulin tolerance test; JNK, Jun N-terminal kinase; MASLD, metabolic dysfunction-associated liver disease; MASH, metabolic dysfunction-associated steatohepatitis; MCD, methionine-choline-deficient; MST4, mammalian sterile 20-like 4; NAFLD, non-alcoholic fatty liver disease; NAS, NAFLD activity score; NASH, non-alcoholic steatohepatitis; NDH, NADH dehydrogenase; NTC, non-targeting control; OXPHOS, oxidative phosphorylation; qRT-PCR, quantitative real-time PCR; SDH, succinate dehydrogenase; si, small interfering; sWAT, subcutaneous white adipose tissue; TAG, triacylglycerol; TH, tyrosine hydroxylase; UCP1, uncoupling protein 1.
