# Supplementary material for: Genetic Ablation of STE20-Type Kinase MST4 Does Not Alleviate Diet-Induced MASLD Susceptibility in Mice
Source: Int J Mol Sci. 2024 Feb 19;25(4):2446. doi: 10.3390/ijms25042446 (PMC10888586; doi:10.3390/ijms25042446)
Supplement: Supplementary file 1 [file ijms-25-02446-s001.zip › ijms-2862732-supplementary.pdf]

Supplementary Figure S1

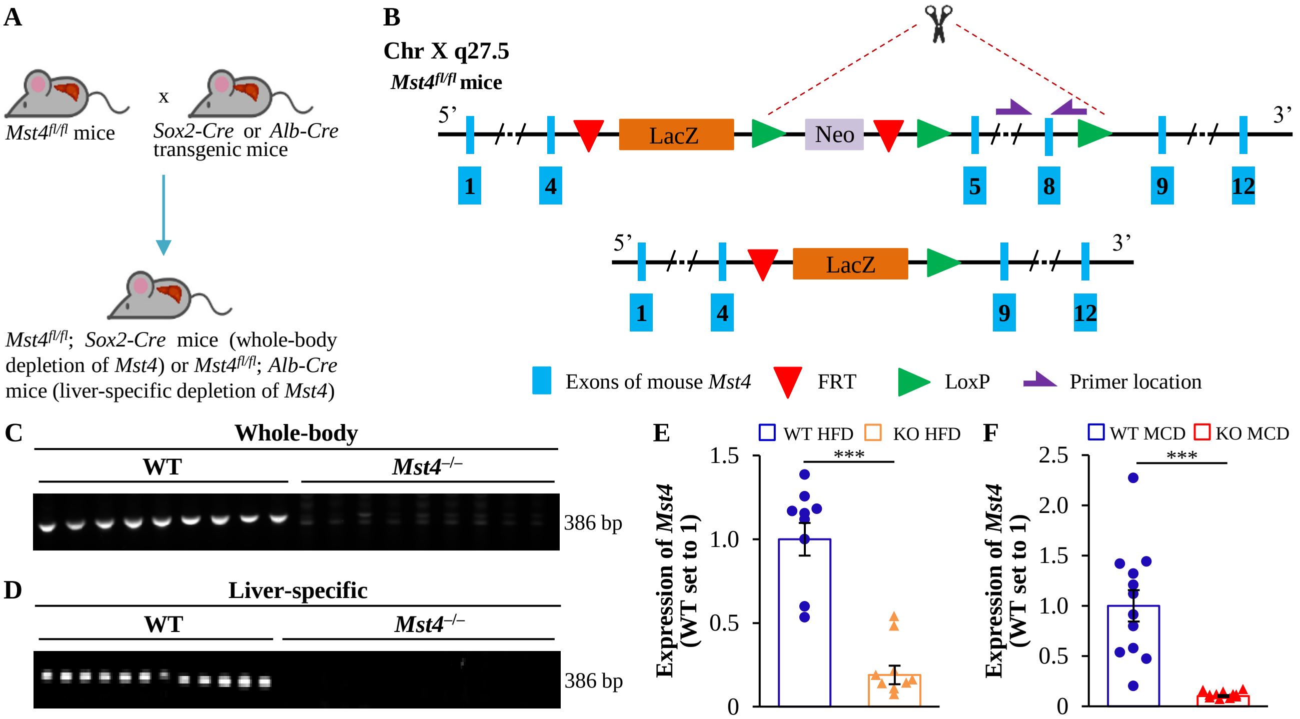

Supplementary Figure S1. Generation and genotyping of whole-body or liver-specific *Mst4* knockout mice. (*A-B*) Schematic diagram depicting the generation of *Mst4* whole-body or liver-specific knockout mice by crossing *Mst4* floxed (*Mst4*$^{fl/fl}$) mice with *Sox2-Cre* or *Alb-Cre* mice, respectively. (*C-D*) Agarose gel electrophoresis of SYBR safe-stained PCR products from the liver tissue of the whole-body (*C*) and liver-specific (*D*) *Mst4*$^{-/-}$ mice and their wild-type littermates using primers shown in (*B*). (*E-F*) Relative *Mst4* mRNA expression was assessed by qRT-PCR in the liver tissue of the whole-body (*E*) and liver-specific (*F*) *Mst4*$^{-/-}$ mice and their wild-type littermates. Data are mean ± SEM from 9-12 mice per group. KO, knockout; WT, wild-type. ***P<0.001

Supplementary Figure S2

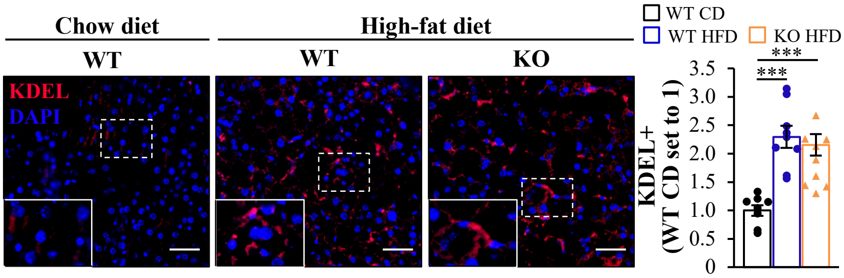

Supplementary Figure S2. Whole-body ablation of MST4 has no effect on the abundance of ER stress marker KDEL in obese mice. Representative liver sections were processed for immunofluorescence with anti-KDEL (red) antibodies; nuclei stained with DAPI (blue). The scale bars represent 25 µm. Quantification of the staining. Data are mean ± SEM from 8-9 mice per group. CD, chow diet; HFD, high-fat diet; KO, knockout; WT, wild-type. ****P*<0.001

Supplementary Figure S3

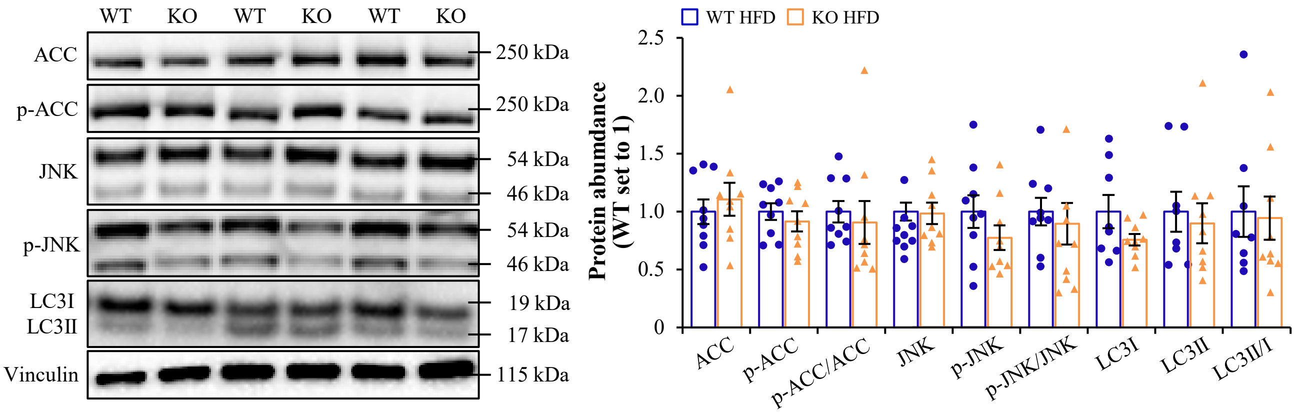

Supplementary Figure S3. Hepatic ACC, JNK, and LC3 signaling is unaffected in high-fat diet-fed *Mst4*$^{-/-}$ mice. Liver lysates were analyzed by Western blot using antibodies specific for ACC, phospho-ACC (Ser^{79}), JNK, phospho-JNK (Thr^{183}/Tyr^{185}), or LC3. Protein levels were measured by densitometry; representative Western blots are shown with vinculin used as a loading control. Data are mean ± SEM from 8-9 mice per group. KO, knockout; WT, wild-type

Supplementary Figure S4

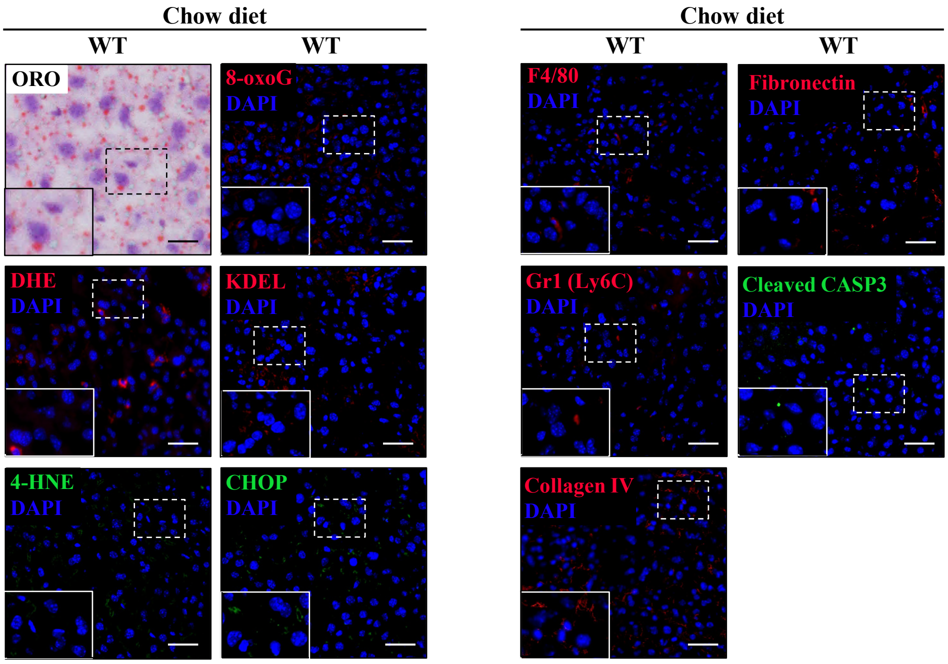

Supplementary Figure S4. Representative liver sections from chow diet-fed wild-type mice were stained with Oil Red O or DHE (red) or processed for immunofluorescence with anti-4-HNE (green), anti-8-oxoG (red), anti-KDEL (red), anti-CHOP (green), anti-F4/80, anti-Gr1 (Ly6C), anti-collagen IV, anti-fibronectin (red), or anti-cleaved CASP3 (green) antibodies; nuclei stained with DAPI (blue). The scale bars represent 25 µm. ORO, Oil Red O; WT, wild-type

Supplementary Figure S5

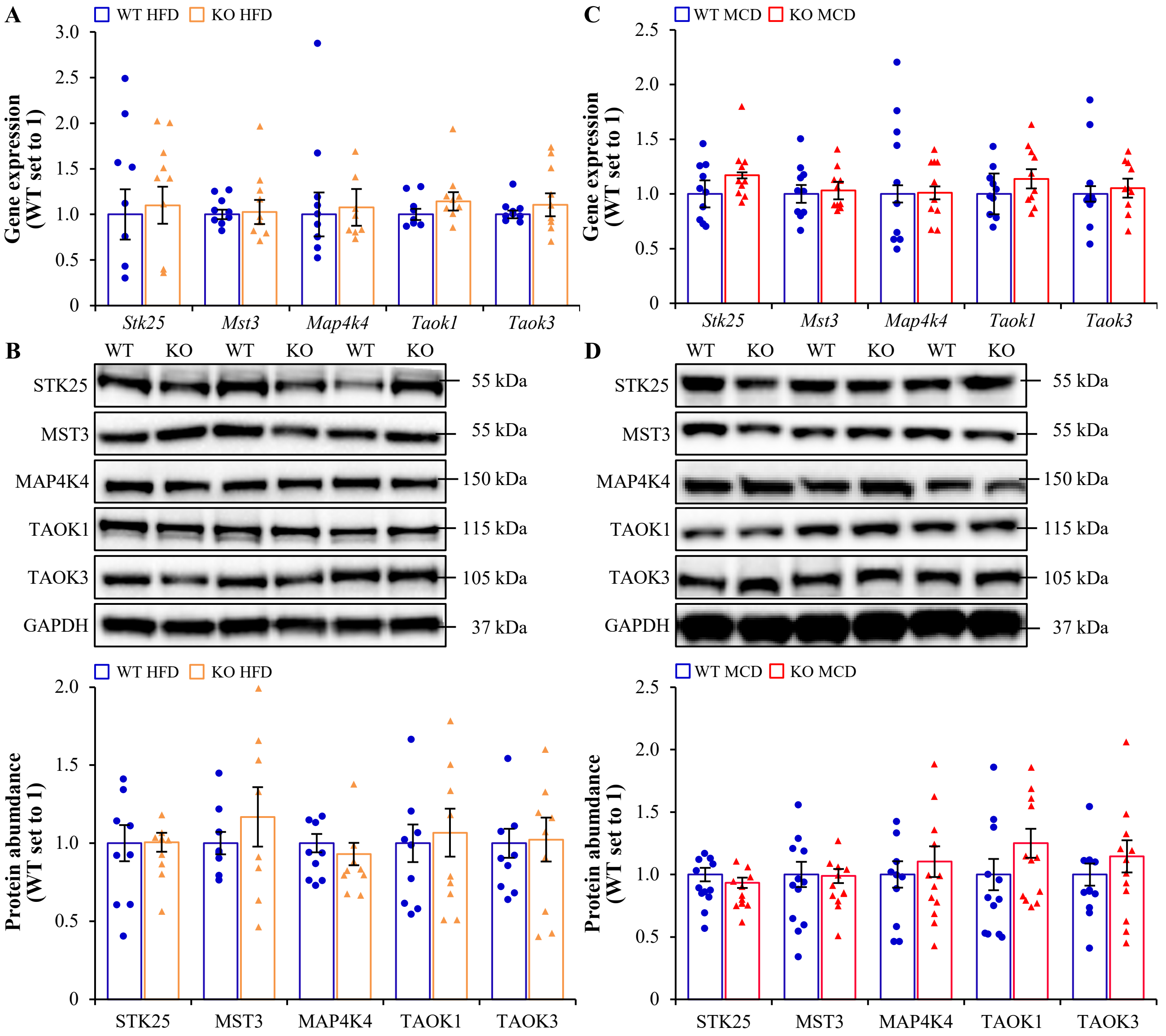

Supplementary Figure S5. Genetic loss of MST4 does not affect the hepatic expression of related STE20-type kinases. (*A-D*) Relative mRNA (*A, C*) and protein (*B, D*) abundance of selected STE20 kinases was assessed by qRT-PCR and Western blot, respectively, in the liver tissue of the whole-body (*A-B*) and liver-specific (*C-D*) *Mst4*$^{-/-}$ mice and their wild-type littermates. (*B, D*) Liver lysates were analyzed by Western blot using antibodies specific for STK25, MST3, MAP4K4, TAOK1, or TAOK3. Protein levels were measured by densitometry; representative Western blots are shown with glyceraldehyde-3-phosphate dehydrogenase (GAPDH) used as a loading control. Data are mean ± SEM from 8-9 mice per group. KO, knockout; WT, wild-type

Supplementary Table S1. List of antibodies used for immunofluorescence/immunohistochemistry and Western blot analysis.

Type	Antibody name and catalog number	Working dilution	Company
Primary antibody	anti-Cytochrome c (MA5-11674)	1:500	Invitrogen (Waltman, MA)
	anti-CHOP (MA1-250)	1:200	Invitrogen
	anti-ACC (#3662)	1:1000	Cell Signaling Technology (Boston, MA)
	anti-p-ACC (#3661)	1:1000	Cell Signaling Technology
	anti-JNK1/2 (#9252)	1:1000	Cell Signaling Technology
	anti-p-JNK1/2 (#4668)	1:1000	Cell Signaling Technology
	anti-LC3 (#2775)	1:1000	Cell Signaling Technology
	anti-vinculin (sc-7269)	1:500	Santa Cruz Biotechnology (Santa Cruz, CA)
	anti-TH (NB300-109)	1:500	Novus Biologicals (Centennial, CO)
	anti-UCP1 (ab10983)	1:500	Abcam (Cambridge, UK)
	anti-total OXPHOS antibody cocktail* (ab110413)	1:2000	Abcam
	anti-4-HNE (ab46545)	1:500	Abcam
	anti-8-oxoG (ab62623)	1:500	Abcam
	anti-KDEL (ab176333)	1:500	Abcam
	anti-F4/80 (MCA497GA)	1:250	Bio-Rad (Hercules, CA)
	anti-Gr1 (Ly6C) (ab15627)	1:300	Abcam
	anti-Collagen IV (NB120-6586)	1:500	Novus Biologicals
	anti-Fibronectin (F3648)	1:300	Sigma-Aldrich (St. Louis, MO)
	anti-Cleaved CASP3 (ab49822)	1:500	Abcam
	anti-αSMA (ab5694)	1:200	Abcam
	anti-STK25 (25821-1-AP)	1:750	Proteintech (Chicago, IL)
	anti-MST3 (#3723)	1:1000	Cell Signaling Technology
	anti-MAP4K4 (PA5-104232)	1:500	Invitrogen
	anti-TAOK1 (26250-1-AP)	1:750	Proteintech
	anti-TAOK3 (28403-1-AP)	1:500	Proteintech
	anti-GAPDH (#2118)	1:1000	Cell Signaling Technology

*This product is an optimized premixed cocktail which contains 5 mouse antibodies, one each against NDUFB8 (ab110242), SDHB (ab14714), UQCRC2 (ab14745), MTCO1 (ab14705), and ATP5A (ab14748).

Type	Antibody name and catalog number	Working dilution	Company
Secondary antibody	Alexa Fluor-594-labeled anti-rat IgG (A11007)	1:500	Invitrogen
	Alexa Fluor-594-labeled anti-rabbit IgG (A21207)	1:500	Invitrogen
	Alexa Fluor-594-labeled anti-mouse IgG (A11005)	1:500	Invitrogen
	anti-rabbit IgG (#7074)	1:1000	Cell Signaling Technology
	anti-mouse IgG (#7076)	1:1000	Cell Signaling Technology
	anti-rabbit IgG (E0432)	1:300	Dako (Glostrup, DK)

Supplementary Table S2. Adipose tissue depots and liver weights were estimated in absolute values, and related to total body weight, in high-fat diet-fed *Mst4*$^{-/-}$ and wild-type mice.

Parameter	WT HFD	KO HFD
eWAT (g)	0.43 ± 0.03	0.47 ± 0.03
eWAT (% body weight)	0.95 ± 0.04	1.08 ± 0.06
sWAT (g)	0.61 ± 0.19	0.46 ± 0.05
sWAT (% body weight)	1.61 ± 0.81	1.02 ± 0.09
Liver (g)	2.20 ± 0.27	2.06 ± 0.24
Liver (% body weight)	4.75 ± 0.45	4.57 ± 0.35

Data are mean ± SEM from 9 mice per group. HFD, high-fat diet; KO, knockout; WT, wild-type
